# Investor Psychology, Mood Variations, and Sustainable Cross-Sectional Returns: A Chinese Case Study on Investing in Illiquid Stocks on a Specific Day of the Week

**DOI:** 10.3389/fpsyg.2020.00173

**Published:** 2020-02-19

**Authors:** Qianwei Ying, Tahir Yousaf, Qurat ul Ain, Yasmeen Akhtar

**Affiliations:** ^1^Business School, Sichuan University, Chengdu, China; ^2^School of Public Finance and Taxation, Southwestern University of Finance and Economics, Chengdu, China; ^3^Noon Business School, University of Sargodha, Sargodha, Pakistan

**Keywords:** speculative stocks, mood variations, day of the week, sustainable cross-sectional returns, anomalies

## Abstract

This paper uncovers a new finding of sustainable cross-sectional variations in stock returns explained by mood fluctuations across the days of the week. Long/short leg of illiquid anomaly returns are extensively related to the days of the week, and the magnitude of excess returns is also striking [Long leg refers to portfolio deciles that earn higher excess returns. Historical evidence suggests that more illiquid stock earn higher excess returns (Amihud, 2002; Corwin and Schultz, 2012)]. The speculative leg of illiquid anomalies is the long leg (Birru, 2018) [The speculative leg falls into the long leg of anomaly because more illiquid stocks are sensitive to investor sentiment (Birru, 2018)]. Therefore, the long (speculative) leg experiences more sustainable high returns on Friday than the short (non-speculative) leg. At the same time, relatively higher long (speculative) leg returns were witnessed on Friday than Monday with a greater magnitude difference. These cross-sectional variations in illiquid stocks on specific days are consistent with the explanation of the limit to arbitrage. The observed variations in cross-sectional returns are sustained and consistent with plenty of evidence from psychology research regarding the low mood on Monday and high mood on Friday.

## Introduction

Behavioral finance researchers critique traditional finance theories by arguing in the favor of the psychology aspect of investors as a core determinant of asset-pricing research. Therefore, it has been a long-standing area of interest for economists to explore whether investor sentiment affects stock prices or not. There is no role of investor sentiment in the presence of classical finance theory. Instead, classical finance theory argues that the competition amongst rational investors – who make portfolios to diversify the statistical properties – will lead to an equilibrium in which prices are equal to realistically discounted values of expected future cash flows. Here, the cross-sectional expected returns depend solely on the cross-sectional systematic risks (Rasheed et al., 2016; Yang et al., 2019). According to classical finance theory, even if there are some irrational investors, the demands of these investors are compensated by arbitrageurs and therefore have no considerable impact on prices (Ying et al., 2019).

A sentimental hypothesis represents a clear prediction that anomalies reveal variation in returns across days of the week, and earlier studies (Baker and Wurgler, 2006, 2007, 2012) have focused on the anomalies that theorizes that prediction is related to sentiment. Particularly, this research has focused on anomalies related to illiquidity and a theory that predicts that one leg should be clearly speculative and one clearly non-speculative. Importantly, individual action and behavior is determined by mood, and it is one of the powerful determinants. Variations in mood have been found to persuade less than fully rational behavior of financial markets, not only from individual investors but from institutional investors as well (Goetzmann et al., 2015).

The weekend effect has not existed since 1975 (Robins and Smith, 2016). The presence of a strong cross-sectional effect is still not surprising since mood variations provide clear predictions, but these patterns do not lead to comprehensive predictions. As Baker and Wurgler (2007) argue in relation to sentiment, theory does not provide obvious comprehensive predictions. For example, speculative stocks are more sensitive to sentiment, and, with the decrease in sentiment, the price of the stocks will also decline. This scenario can lead to fluctuations in quality, which will cause an increase in prices of non-speculative or safe stocks (Kong et al., 2019). Therefore, sentiment provides obvious cross-sectional prediction as has previously been argued. This study will focus on specific types of cross-sectional investment strategies, which will clearly show day-of-the-week return by considering the sentimental hypothesis.

In earlier studies, many researchers documented that stock markets perform low on Mondays [early studies include (Cross, 1973; French, 1980; Gibbons and Hess, 1981)]. Though many studies explored the weekend effect, none of them have produced satisfactory results. Investor sentiment diverges across the days of the week since mood is a deviating factor that affects sentiment (Ma and Tanizaki, 2019). In capital markets, the existence of pessimism and optimism (which is not related to the fundamentals) – generally called sentiment – provides clear predictions of cross-sectional return. The variation in sentiment will have a contemporary effect on returns, and it will highly affect the prices of stocks that are not easy to value, that are very subjective to value, or that are difficult to arbitrage (Baker and Wurgler, 2006). Therefore, the hypothesis predicts that, in comparison to non-speculative stocks, speculative stocks will earn high returns on Friday and low returns on Monday (Birru, 2018).

The analysis of the variation in mood across the days of the week has remained a vigorous research dimension in the field of psychology ever since the first extensive study was conducted by Rossi and Rossi (1977). Though the exact flow of the variations in mood over the course of the week has long been debated, one comparatively unquestionable finding has been discovered in the literature: there exists a higher mood on the weekend and Friday than from Monday to Thursday. Generally, the mood increases from Thursday to Friday and decreases on Monday. Mixed results exist in literature regarding the mood variation from Monday to Thursday (Shah et al., 2019). Some recent studies have used a large heterogeneous sample of the individuals and expanded our understandings. For example, Stone et al. (2012) and Helliwell and Wang (2014) used the sample data from the Gallup organization of the United States that was gathered through telephonic questionnaires, and this study consisted of more than 340,000 individuals above the age of 18. Their findings are also consistent with the theory that mood is higher on Friday than Monday to Thursday. As only Friday and Monday are the days of the week that provide the obvious psychological forecast, our analysis will focus only on these days. The strong psychological evidence that mood is higher on Friday and lower on Monday predicts higher returns for speculative stocks on Friday than non-speculative characteristics of stocks, and an inverse pattern exists on Monday. Our study contributes to the literature: it presents a specific explanation of stocks as being sensitive to sentiment, and it provides evidence of cross-sectional variations of returns on particular days by linking a speculative leg of illiquid stocks with mood theory from psychology literature. This study also provides different investment strategies to earn excess returns across the days of the week by investing in illiquid stocks.

Numerous hypotheses motivate the analysis in this study. One of the possible theories is that the trading behavior of institutions changes with the days of the week, and this, in turn, causes the predictable variation of cross-sectional returns across the days of the week. Other reasons are related to the content and timing of the news release. Cross-sectional variation may be found in the contents and timings of the announcement of good or bad news. Another possible explanation is related to the timing of macroeconomic news announcements; it is sometimes observed that good or bad macroeconomic news is systematically released on particular days of the week. These systematic patterns have cross-sectional return effects, and this study has incorporated these explanations in order to check and verify the true relationship.

On the basis of the published literature we test four hypotheses.

H1: The speculative leg of anomalies earns a higher return on Friday than Monday due to mood variations across the days of the week.
H2: The speculative leg of anomalies earns higher stock returns on Friday than the non-speculative leg.
H3: The speculative leg of anomalies earns higher long minus short strategy returns on Friday than Monday.
H4: The observed cross-sectional variation in the stock return is inconsistent with the impact of Firm-specific news and Macroeconomic news.

## Materials and Methods

In this section, our analysis has focused on the characteristics of illiquid stocks that theory predicts are affected by the sentiment. According to Baker and Wurgler (2006, 2007), stocks that are most affected by the sentiment are difficult to value or are subjective and hard to arbitrage. Practically, stocks that have either subjective characteristics in terms of valuation or that are hard to arbitrage are likely to be the same (Birru, 2018).

Literature from Psychology hypothesizes that mood affects decisions when situations are not clear or adequate information is not available (Clore et al., 1994; Forgas, 1995; Hegtvedt and Parris, 2014; Sarfraz et al., 2019). Conversely, stocks that do not have a precise valuation will give experienced investors a misrepresentation of the valuation of the stocks, and this varies with the existing state of sentiment. Baker and Wurgler (2006) have assessed the related dimensions to differentiate the speculative intensity of stocks, and they found that stocks are the paying status of dividend, intense growth, size, level of distress, profitability, and age. More precisely, Birru (2018) mentioned that illiquid stocks face greater impediments to arbitrage. Therefore, our analysis will focus on illiquid stocks because these stocks are sensitive to sentiment, and they should have higher speculative returns on Friday than Monday. For this purpose, we have taken two measures for illiquid stocks: Amihud’s illiquidity measure (Amihud, 2002) and the Bid-Ask spread (Corwin and Schultz, 2012)^1^. Portfolio performance is measured through the value of the Jensen Alpha, and it is a risk-adjusted measure of performance that represents the average return of the portfolio investment, below or above the different asset pricing models, given investment’s beta or market average return.

### Portfolio Construction

Illiquidity is measured following the methodology of Amihud (2002) and Corwin and Schultz (2012). Portfolios are then generated by making 10 deciles based on calculated values for each stock following the methodology of Amihud (2002) and Corwin and Schultz (2012). We took, however, only decile one and 10 of both measures for the portfolio construction, as our analysis was based on speculative and non-speculative characteristics of each portfolio and fell only in extreme deciles. Portfolios constructed for both anomalies are rebalanced every month. A penalized expected risk criterion is one of the widely used portfolio construction approach (Luo et al., 2019). Birru (2018) mentioned that illiquid stocks are sensitive to sentiment, and stocks that are more illiquid face higher impediments to arbitrage. Therefore, the highest decile of illiquid stocks will earn higher returns on Friday than Monday and higher long minus short returns on Friday than Monday. Supplementary Table S1 provides more insights regarding the possible returns on particular days and the details of both anomalies.

Supplementary Table S1 describes the division of the sample for anomalies and speculative strategies. It indicates the division of anomalies into Long leg and Short leg, and it also indicates the expected speculative leg for each anomaly and the brief explanation for speculative reason. The table also reports the expected returns for a speculative leg on particular days.

### Data

The data set that we use for our analysis was taken from Wind Information Incorporation^2^. The analysis period for our study was from January 1996 to December 2018 for Amihud’s illiquidity measure and from 2005 for the Bid-Ask spread measure and the target of our analysis is Chinese A-shares market for both the Shanghai and Shenzen stock exchanges. The Chinese A-shares market started domestic trading in 1990 with the establishment of both stock exchanges.

Our focus was based on post-1996 data for two reasons. The first reason was to ensure uniformity in the data. Though principles of fair trade and reporting were introduced in 1993, companies do not have much guidance on how to do this practically. The implementations of rules and regulations take time and yet produced a lot of discrepancies in the early years. Most of the firms take the liberty to make their standards of implementation for financial reporting, and this thus creates comparability issues (Ghulam et al., 2019). The second reason belongs to the minimum required numbers in the creation of portfolios. To attain reasonable power and precision, the portfolio construct should be based on 10 equal deciles, and each decile should have a minimum of 50 values after using all filters.

## Results and Discussion

### Results

#### Illiquidity: Friday Long Minus Short

Focusing on the long minus short returns on Friday for Amihud’s illiquidity measure, panel A of Table 1 shows that Friday accounts for more than 113 basis points per month excess returns according to CAPM, while the Fama and French three-factor alpha, Carhart four-factor alpha, and Fama and French five-factor alpha account for 95, 79, and 75 basis points of excess returns, respectively, for each month. Panel B of Table 1, however, examines long minus short returns for portfolio constructs through Amihud’s illiquid measure on Monday. According to the results of Table 2, Monday accounts for negative alpha values for all measurement models, and these results are consistent with the mood theory that Friday accounts for higher long minus short strategy returns due to a higher mood than the lower mood on Monday.

**TABLE 1:** Panels **A** and **B**: long minus short strategy returns (Amihud’s illiquidity measure).

Panel A (Friday long minus short strategy returns)						

	Friday long	*T*-statistics	Friday short	*T*-statistics	Friday long–short	*T*-statistics
CAPM	0.0113672	(2.91)	–0.0000811	(−2.16)	0.0113662	(3.15)
FF3	0.0094585	(2.40)	–0.000114	(−2.62)	0.0095726	(2.45)
Carhart4	0.0077422	(2.49)	–0.0000739	(−2.14)	0.0079358	(2.92)
FF5	0.0132384	(2.69)	–0.0000167	(−2.03)	0.0074951	(2.87)

**Panel B (Monday Long minus short strategy returns)**						

	**Monday long**	***T*-statistics**	**Monday short**	***T*-statistics**	**Monday long–short**	***T*-statistics**

CAPM	–0.0004678	(−3.35)	0.0012095	(3.05)	–0.0016774	(−2.29)
FF3	–0.0011018	(−2.81)	0.0010962	(2.84)	–0.002198	(−2.69)
Carhart4	–0.0013681	(−2.00)	0.0009015	(2.50)	–0.0022696	(−2.71)
FF5	–0.0018303	(−2.34)	0.0009323	(1.91)	–0.0027626	(−3.09)

*This table examines long minus short monthly portfolio returns based on Amihud’s illiquidity measure to invest in a particular day of the week. Panel A reports long minus short strategy returns on Friday, and panel B reports long minus short strategy returns on Monday. The panels indicate the values of Alpha for CAPM, the Fama and French three-factor model, the Carhart four-factor model, and the Fama and French five-factor model. Portfolios are equally weighted, and values of *t*-statistics are adjusted for autocorrelation and heteroscedasticity.*

**TABLE 2:** Panels **A** and **B**: long minus short strategy returns (Bid-Ask spread measure).

Panel A (Friday long minus short strategy returns)						

	Friday long	*T*-statistics	Friday short	*T*-statistics	Friday long–short	*T*-statistics
CAPM	0.0024509	(3.01)	−0.0022354	(−5.30)	0.0046863	(6.34)
FF3	0.0023808	(2.88)	−0.0025514	(−5.99)	0.0049322	(6.60)
Carhart4	0.0022996	(2.81)	−0.0025135	(−5.81)	0.0048096	(6.35)
FF5	0.0026698	(3.27)	−0.0023119	(−5.31)	0.0044491	(5.87)

**Panel B (Monday long minus strategy returns)**						

	**Monday long**	***T*-statistics**	**Monday short**	***T*-statistics**	**Monday long–short**	***T*-statistics**

CAPM	0.000831	(2.18)	−0.0021325	(−4.15)	0.0029635	(5.44)
FF3	0.0008291	(2.15)	−0.0024029	(−4.45)	0.0032319	(5.91)
Carhart4	0.0004254	(2.59)	−0.0023575	(−4.30)	0.0027829	(5.24)
FF5	0.000181	(2.25)	−0.0023689	(−4.26)	0.0025499	(4.81)

*This table examines long minus short monthly portfolio returns based on Bid-Ask spread (Corwin and Schultz, 2012) to invest in a particular day of the week. Panel A reports long minus short strategy returns on Friday, and panel B reports long minus short strategy returns on Monday. The panels report the values of Alpha for CAPM, the Fama and French three-factor model, the Carhart four-factor model, and the Fama and French five-factor model. Portfolios are equally weighted, and values of *t*-statistics are adjusted for autocorrelation and heteroscedasticity.*

Panel A of Table 2 focuses on the long minus short strategy returns of Bid-Ask spread anomaly on Friday and presents the same predication that Friday alone provides 46, 49, 48, and 44 basis points monthly in excess returns against CAPM, the Fama and French three-factor model, the Carhart four-factor model, and the Fama and French five-factor alpha, respectively. Meanwhile, panel B of Table 3 is also consistent with mood prediction and provides comparatively lower long minus short strategy returns on Monday than Friday. The magnitude difference between Friday and Monday portfolio strategy returns are much higher for Amihud’s illiquid measure, whereas the Bid-Ask spread anomaly provides almost double long minus short strategy returns on Friday than Monday.

**TABLE 3:** Panels **A** and **B**: Friday minus Monday strategy returns (Amihud’s illiquidity measure and Bid-Ask spread measure).

Panel A (Friday minus Monday strategy returns of Amihud’s illiquidity measure)						

	Friday long–short	*T*-statistics	Monday long–short	*T*-statistics	Friday–Monday	*T*-statistics
CAPM	0.0113662	(3.15)	–0.0016774	(−2.29)	0.0130436	(3.48)
FF3	0.0095726	(2.45)	–0.002198	(−2.69)	0.0117706	(3.09)
Carhart4	0.0079358	(2.92)	–0.0022696	(−2.71)	0.0102054	(2.67)
FF5	0.0074951	(2.87)	–0.0027626	(−3.09)	0.0102577	(2.62)

**Panel B (Friday minus Monday strategy returns of Bid-Ask spread measure)**						

	**Friday long–short**	***T*-statistics**	**Monday long–short**	***T*-statistics**	**Friday–Monday**	***T*-statistics**

CAPM	0.0046863	(6.34)	0.0029635	(5.44)	0.0017229	(2.17)
FF3	0.0049322	(6.60)	0.0032319	(5.91)	0.0017003	(2.10)
Carhart4	0.0048096	(6.35)	0.0027829	(5.24)	0.0020267	(2.49)
FF5	0.0044491	(5.87)	0.0025499	(4.81)	0.0018993	(2.27)

*This table examines Friday minus Monday monthly portfolio returns based on Amihud’s illiquidity measure (Panel A) and the Bid-Ask spread measure (Panel B) to invest on a particular day. The table reports the values of Alpha for CAPM, the Fama and French three-factor model, the Carhart four-factor model, and the Fama and French five-factor model. Portfolios are equally weighted, and values of *t*-statistics are adjusted for autocorrelation and heteroscedasticity.*

#### Friday Minus Monday Strategy Returns

Table 3 presents more direct estimations of the sentimental hypothesis by comparing Friday long minus short strategy returns and Monday long minus short strategy returns. The magnitude difference between both days varies from 102 basis points to 130 basis points for all estimations. For instance, by observing the CAPM alpha of Amihud’s illiquidity measure from Panel A, Fridays account for more than 132 basis points excess returns for each month, and it means that by following this strategy investors can gain a 15.84% yearly excess return. Results for the Bid-Ask spread anomaly are also consistent with the theory, and Friday earns higher monthly strategy returns than Monday. Our results are not only striking in terms of magnitude, but our results are consistent with the findings of mood theory that Friday sustained higher returns in comparison to Monday, and these findings are consistent for both anomalies, and results are presented in Panel A and B of Table 3.

#### Asymmetry in Long Leg

Panels A and B of Table 4 examine the returns difference of the long leg between Friday and Monday for both anomalies. The mispricing story based on sentimental hypothesis gives a prediction of asymmetry when comparing the Friday long leg with Monday’s long leg. An explanation based on the sentiment explains that the displayed return trend should be endorsable to the speculative leg. Therefore, panel A and B show only the long leg for both anomalies since the speculative leg is the long leg for both measures. The return difference in long leg portfolios for both days is solely larger than the return of the long minus short portfolio. For example, focusing on the CAPM alpha of the Friday long minus the Monday long for the portfolio based on Amihud’s illiquidity measure leads to an increase of 118 excess basis points strategy returns on Friday over Monday. To give a more straightforward explanation for this investment strategy, we can say that investors can earn 118 basis points excess returns by merely investing in the long leg of the portfolio based on Amihud’s illiquid measure for only two days (take the long position on Friday, shorten it on Monday, and the rest of the days can be used to invest in risk-free investment).

**TABLE 4:** Panels **A** and **B**: asymmetry in long leg (Amihud’s Illiquidity measure and Bid-Ask spread measure).

Panel A (asymmetry in long leg of Amihud’s illiquidity measure)						

	Friday long	*T*-statistics	Monday long	*T*-statistics	Friday long–Monday long	*T*-statistics
CAPM	0.0113672	(2.91)	–0.0004678	(−3.35)	0.011835	(3.13)
FF3	0.0094585	(2.40)	–0.0011018	(−2.81)	0.0105604	(2.75)
Carhart4	0.0077422	(2.49)	–0.0013681	(−2.00)	0.00923	(2.38)
FF5	0.0132384	(2.69)	–0.0018303	(−2.34)	0.0093422	(2.36)

**Panel B (asymmetry in long leg of Bid-Ask spread measure)**						

	**Friday long**	***T*-statistics**	**Mondaylong**	***T*−statistics**	**Friday long–Monday long**	***T*-statistics**

CAPM	0.0024509	(3.01)	0.000831	(2.18)	0.0024189	(1.51)
FF3	0.0023808	(2.88)	0.0008291	(2.15)	0.0023063	(1.42)
Carhart4	0.0022996	(2.81)	0.0004254	(2.59)	0.002154	(1.30)
FF5	0.0026698	(3.27)	0.000181	(2.25)	0.0018154	(1.09)

*This table examines Friday monthly portfolio returns based on Amihud’s illiquidity measure (Panel A) and the Bid-Ask spread measure (Panel B) to invest in the long leg on a particular day. The table reports the values of Alpha for CAPM, the Fama and French three-factor model, the Carhart four-factor model, and the Fama and French five-factor model. Portfolios are equally weighted, and values of *t*-statistics are adjusted for autocorrelation and heteroscedasticity.*

#### Robustness Test

##### Macroeconomic news effect

It is unlikely that good or bad news has a systematic pattern of being announced on a particular day of the week, but it is possible that a cross-sectional effect is generated due to these macroeconomic news announcement effects. For instance, illiquid stocks are sometimes more sensitive toward these announcements than others. Therefore, we gathered data on the monthly macroeconomic announcement dates by following (Savor and Wilson, 2013) and took the announcement dates data of the CPI (Consumer Price Index) and PPI (Producer Price Index). We focused on the days when these figures are released. Panel A and B of Supplementary Table S2 provide results of strategy returns for both anomalies when returns of particular dates are excluded from the sample. The results indicate that earlier patterns of cross-sectional returns for Friday and Monday are robust to the exclusion of macroeconomic announcement dates. Thus, the results are not consistent with the explanation that the observed cross-sectional pattern is due to the impact of macroeconomic news announcements.

##### Firm-specific news impact

A possible explanation for this cross-sectional effect on a particular day could be the non-random timing of firm-specific news announcements (Guo and Huang, 2019). Therefore, to make this argument valid, speculative and non-speculative firms should have a systematic difference in the announcements of good and bad news. To verify this argument, we took data for announcement dates of earning and dividend declarations at the firm level. Literature has suggested that earning announcement dates are off by some days (Dellavigna and Pollet, 2009). We excluded two days before the announcement and two days post-announcement by considering the conservative approach. This approach was beneficial because a week has five working days, and the exclusion of *t* − 2 and *t* + 2 days ensured equal elimination for each day of the week. Panel A and B of Supplementary Table S3 examine the results, with the exclusion of *t* − 2 to *t* + 2 dates, of earnings and dividend announcements. The results indicate that the magnitude difference of strategy returns has no significant change, and findings of both anomalies are not consistent with the explanation that cross-sectional variation of returns on Friday and Monday is derived by firm-specific news.

##### Impact of institutional ownership

Firms with high institutional ownership are expected to be less effected by sentiment, and, at the same time, firms with less institutional ownership and high individual ownership structures are more sensitive to sentiment. Therefore, we have further divided existing portfolios on the basis that institutional investment and firms that fall below the median provide a robust explanation: cross-sectional variation in stock returns is higher for stocks that have less institutional ownership because stocks with less institutional ownership are prone toward investor sentiment. Supplementary Table S4 provides only the results for both anomalies with the firms that fall below the median value of institutional ownership. The results indicate that firms with low institutional investment and more illiquid are more sensitive to sentiment and provide higher returns on Friday than Monday.

### Discussion

The literature of psychology predicts and gives robust findings related to mood elevation on Friday over Monday, and this causes higher returns for speculative stocks on Fridays. These robust findings also predict that returns will be relatively low on Monday in parallel with the decreasing mood on Monday. Hence, a new strategy is emerging with the prediction that anomaly returns will be higher on Fridays for those where the speculative leg is the long leg. Results have confirmed the prediction that was found in the data and examined long minus short returns for Amihud’s illiquid measure and the Bid-Ask spread anomaly on Friday and the long minus short returns of illiquid anomaly on Monday. Striking results emerged when the Friday minus Monday estimation provided higher alpha values for the portfolio constructed on the basis of Amihud’s illiquid measure for all models. The results were not only striking in terms of magnitude, but they were also consistent with the findings of mood theory that Friday sustained higher returns in comparison to Monday, and these findings were consistent for both anomalies.

The findings of difference in the long leg for both anomalies were also consistent with the sentiment, based on investor mood, that the day of the week effect prevails in cross-sectional returns for the speculative leg of the portfolio investment. Therefore, it was again confirmed that the speculative leg earns higher returns on Friday than on Monday for the same speculative leg. Our findings were also in accordance with the explanation that the observed pattern of the cross-sectional effect on a particular day was not explained by the non-random timing of firm-specific news announcements, and they were inconsistent with the finding that the cross-sectional effect is generated due to these macroeconomic news announcement effects, as it is unlikely that good or bad news had a systematic pattern to be announced on a particular day of the week. We have also performed an additional robust test by dividing the firms into two sections on the basis of institutional ownership, and our results were again consistent with the portfolio returns of speculative stocks. The findings indicate that Friday earns higher stock returns for the portfolio of the firms that fall in the lower median because firms that have less institutional investment (and thus more individual ownership) are more prone to sentiment.

## Conclusion

The study has found a strong, predictable cross-sectional variation in illiquid stocks across the days of the week. Although the Chinese market has a different investment culture and political environment, our results are consistent with the findings of Birru (2018). The study found that the speculative leg of illiquid stocks earned higher returns on Friday than Monday in comparison to non-speculative stocks. Our results are also concurrent with firm-specific news announcements, macroeconomic news announcements, and monthly portfolio returns.

Psychology literature has found a consistent variation in mood across the days of the week, where mood increases on Friday and decreases on Monday. Our results regarding the cross-sectional pattern in illiquid stocks were consistent with the psychology findings, and returns are relatively higher on Friday when the mood is higher and lower on Monday when the mood is lower. Moreover, the study provides different strategies to earn excess returns across the days of the week by investing in illiquid stocks. The findings of the paper are an extension of the evidence of Baker and Wurgler (2012) that during the high-sentiment time investors tend to have low demand for safe investments, whereas, during the low sentiment time, investors tend to have a flight toward quality. Our study gives a more specific explanation that stocks are sensitive to sentiment, and we provide evidence of cross-sectional variations of returns on particular days by linking the speculative leg of illiquid stocks with mood theory from psychology literature. Additionally, our research will help academicians and practitioners in making investment strategies for investment or future research.

There are several limitations to the study: this study is based upon a Chinese dataset, and the structure of the Chinese stock market is quite different to that of the rest of the world. Therefore, the results of the study may not be generalized to other markets. The Chinese market has a strong government influence, and this study can be further tested by segregating the state-owned and non-state-owned enterprises. For future directions, this research can also be expanded by incorporating the different dimensions of emotions that affect an individual’s mood, e.g., valence, arousal, state-related, and trait-related emotions.

## Data Availability Statement

The raw data supporting the conclusions of this article will be made available by the authors, without undue reservation, to any qualified researcher.

## Author Contributions

QY supervised this study and involved with the methodology section, data collection, and made revisions to the manuscript. TY performed the formal analysis, the methodology of the manuscript, and applied techniques through software. QA wrote, reviewed, and edited the draft, and helped with the data collection. YA proofread the manuscript.

## Conflict of Interest

The authors declare that the research was conducted in the absence of any commercial or financial relationships that could be construed as a potential conflict of interest.

## Footnotes

1 The estimation method of anomalies and their definitions are provided in the Supplementary Material.

2 The wind is the most extensive and most reliable data provider database of China. The wind database serves more than 70% of QFII (Qualified Foreign Institutional Investors) and 90% of Chinese financial institutes.
