# Supplementary material for: Investor Psychology, Mood Variations, and Sustainable Cross-Sectional Returns: A Chinese Case Study on Investing in Illiquid Stocks on a Specific Day of the Week
Source: Front Psychol. 2020 Feb 19;11:173. doi: 10.3389/fpsyg.2020.00173 (PMC7043266; doi:10.3389/fpsyg.2020.00173)
Supplement: Supplementary file 1 [file Data_Sheet_1.docx]

Supplementary Material

# Definitions and Estimations of Anomalies

### Amihud’s Illiquidity Measure

(Amihud 2002) finds that high illiquid stocks outperform the stocks that are less illiquid. Amihud’s illiquidity measure is the most widely used measure for stock illiquidity in finance literature. It mainly used to measure the degree of illiquidity of stocks, and it was proposed by YakovAmihud in 2002. Historically, stocks with higher illiquidity value tend to generate higher returns and it means that speculative leg will be long leg of the anomaly. Where illiquidity measure as

$\mathbf{ILLIQ}_{\mathbf{it}}\mathbf{=}\frac{\mathbf{1}}{\mathbf{I}}\sum_{\mathbf{t=1}}^{\mathbf{I}} \left| \mathbf{R}_{\mathbf{it}} \right|\mathbf{/}\mathbf{V}_{\mathbf{it}}\mathbf{P}_{\mathbf{it}}$**(1)**

Here, $\mathbf{I}$ is the number of days. $\boldsymbol{R}_{\boldsymbol{it}}$is the return on a daily basis for time t and firm i, $\boldsymbol{V}_{\boldsymbol{it}}$ is the respective daily volume and $\boldsymbol{P}_{\boldsymbol{it}}$ is the price of stock i for time period t. Through the mentioned formula, we have measured the illiquidity of each firm.

### Bid-Ask Spread

(Corwin and Schultz 2012) has given another measure of illiquidity called Bid-Ask spread. This measure based on daily high low prices spread, and the rationale behind the measure is high prices are buyers initiated, and low are seller initiated. The measure presents not only bid-ask spread but also reflect stock variance. Volatility is proportionate to return interval whereas the spread remains constant for shorter horizon. Based on given explanation, (Corwin and Schultz 2012) develop the estimator as follows:

$\boldsymbol{\beta=E}\left[ \boldsymbol{\sum}_{\mathbf{j=0}}^{\mathbf{1}}\left[ \mathbf{ln} \right.\mathbf{(}\frac{\mathbf{H}_{\mathbf{t+j}}^{\mathbf{0}}}{\mathbf{L}_{\mathbf{t+j}}^{\mathbf{0}}}\mathbf{)}\mathbf{]}^{\mathbf{2}} \right]$ **(2)**

Here $\boldsymbol{\beta}$ is the expected total of price ranges for two successive days. $\boldsymbol{H}_{\boldsymbol{t}}^{\boldsymbol{0}}$is the highest price on time t and $\boldsymbol{L}_{\boldsymbol{t}}^{\boldsymbol{0}}$ is the lowest price on time t.

Now, γ will represent the range of high/low ratio of the two days. Expressions will be as follows:

$\boldsymbol{\gamma}_{\mathbf{t}}\mathbf{=}\left[ \mathbf{ln}\left( \left. \frac{\mathbf{H}_{\mathbf{t-1,t}}^{\mathbf{o}}}{\mathbf{L}_{\mathbf{t-1,t}}^{\mathbf{o}}} \right) \right. \right]^{\mathbf{2}}$ **(3)**

Using the estimated values of $\boldsymbol{\beta}$and$\boldsymbol{\gamma}$, the proportionate difference between both values of $\boldsymbol{\gamma}_{\boldsymbol{t}}$ and $\boldsymbol{\beta}_{\boldsymbol{t}}$ used to derive the following:

$\boldsymbol{\alpha}_{\mathbf{t}}\mathbf{=}\frac{\sqrt{\boldsymbol{2\beta}_{\mathbf{t}}}\mathbf{-}\sqrt{\boldsymbol{\beta}_{\mathbf{t}}}}{\mathbf{3-2}\sqrt{\mathbf{2}}}\mathbf{-}\sqrt{\frac{\boldsymbol{\gamma}_{\mathbf{t}}}{\mathbf{3-2}\sqrt{\mathbf{2}}}}\mathbf{=(1+}\sqrt{\mathbf{2}}\mathbf{)(}\sqrt{\boldsymbol{\beta}_{\mathbf{t}}}\mathbf{-}\sqrt{\boldsymbol{\gamma}_{\mathbf{t}}}\mathbf{)}$ **(4)**

The value of $\boldsymbol{\alpha}$ will have time variations because it is estimated from the high and low values and it represents the difference between the adjustment of a single day and two-day period. Following the last equation, the value of the bid-ask spread is given as follows.

$\mathbf{S=}\frac{\mathbf{2(}\mathbf{e}^{\boldsymbol{\alpha}_{\mathbf{t}}}\mathbf{-1)}}{\mathbf{1+}\mathbf{e}^{\boldsymbol{\alpha}_{\mathbf{t}}}}$ **(5)**

Here $\mathbf{S}$ is the spread, $\mathbf{e}$ is mathematical constant and value of $\boldsymbol{\alpha}$ is taken from equation 4.

# Supplementary Figures and Tables

**Table S1:** Description of Anomalies and speculative investment strategies

|  | **Amihud’s Illiquidity** | **Bid-Ask Spread** |
| --- | --- | --- |
| **Long Leg** | Decile 10 | Decile 10 |
| **Short Leg** | Decile1 | Decile 1 |
| **Speculative Leg** | Decile 10 | Decile 10 |
| **Predicted Speculative Leg Return on Friday** | Higher | Higher |
| **Predicted Speculative Leg Return on Monday** | Lower | Lower |
| **Explanation(Why Speculative)** | Limit to Arbitrage | Limit to Arbitrage |

**Table S2 Panel A & B:** Excluding Macro news announcement dates

**Table S2 Panel A**

|  | **Friday Long-Short** | **T statistics** | **Monday Long-Short** | **T statistics** | **Friday - Monday** | **T statistics** |
| --- | --- | --- | --- | --- | --- | --- |
| **CAPM** | .0082662 | (3.87) | -.0019254 | (-2.12) | .010929 | (3.94) |
| **FF3** | .007726 | (3.23) | -.002488 | (-2.19) | .009785 | (3.33) |
| **Carhart4** | .0071358 | (2.96) | -.0029766 | (-2.69) | .010121 | (3.09) |
| **FF5** | .0067951 | (2.52) | -.0031826 | (-3.12) | .009981 | (2.93) |

**Table S2, Panel B**

|  | **Friday Long-Short** | **T statistics** | **Monday Long-Short** | **T statistics** | **Friday - Monday** | **T statistics** |
| --- | --- | --- | --- | --- | --- | --- |
| **CAPM** | .0042584 | (4.75) | .0026629 | (4.76) | .0016012 | (3.95) |
| **FF3** | .0048249 | (5.81) | .0030512 | (5.27) | .0027991 | (3.81) |
| **Carhart4** | .0041625 | (5.89) | .0024290 | (4.98) | .0017412 | (4.15) |
| **FF5** | .0040270 | (5.21) | .0022972 | (4.21) | .0017327 | (4.22) |

Table S2 examines Long minus Short &Friday minus Monday monthly portfolio returns based on Amihud’s illiquidity measure (Panel A) & Bid-Ask spread measure (Panel B) to invest on a particular day. The table reports the values of Alpha for CAPM, Fama & French 3 factor Model, Carhart 4 factor model, and Fama& French 5 factor model. Portfolios are equally weighted, and values of t-statistics are adjusted for autocorrelation & heteroscedasticity.

**Table S3 Panel A & B:** Excluding Firm Specific news announcement dates

**Table S3 Panel A**

|  | **Friday Long-Short** | **T statistics** | **Monday Long-Short** | **T statistics** | **Friday - Monday** | **T statistics** |
| --- | --- | --- | --- | --- | --- | --- |
| **CAPM** | .0085719 | (2.39) | -.0021525 | (-3.19) | .010735 | (4.89) |
| **FF3** | .0078524 | (2.32) | -.0027985 | (-3.51) | .010651 | (4.61) |
| **Carhart4** | .0072926 | (2.14) | -.0032649 | (-2.88) | .0105631 | (4.36) |
| **FF5** | .0070791 | (2.02) | -.0032990 | (-3.12) | .010285 | (4.49) |

**Table S3, Panel B**

|  | **Friday Long-Short** | | | **T statistics** | | **Monday Long-Short** | | **T statistics** | | **Friday - Monday** | | **T statistics** |
| --- | --- | --- | --- | --- | --- | --- | --- | --- | --- | --- | --- | --- |
| **CAPM** | .0045662 | | | (3.81) | | .0024321 | | (3.78) | | .0021101 | | (2.58) |
| **FF3** | .0051397 | | | (3.97) | | .0028590 | | (3.27) | | .0022812 | | (2.37) |
| **Carhart4** | .0042519 | | | (4.12) | | .0022126 | | (3.81) | | .0020909 | | (2.71) |
| **FF5** | .0042993 | | | (3.92) | | .0021954 | | (3.42) | | .0021392 | | (2.89) |
| **FF5** | | .0315236 | (12.87) | | -.0094985 | | (-4.42) | | .0410222 | | (12.56) | |

Table S3 examines Long minus Short & Friday minus Monday monthly portfolio returns based on Amihud’s illiquidity measure (Panel A) & Bid-Ask spread measure (Panel B) to invest on a particular day. The table reports the values of Alpha for CAPM, Fama& French 3 factor Model, Carhart 4 factor model, and Fama& French 5 factor model. Portfolios are equally weighted, and the values of t-statistics are adjusted for autocorrelation & heteroskedasticity.

**Table S4 Panel A & B:** Friday minus Monday Strategy returns (Portfolio returns for Illiquid stocks only with low institutional Ownership stocks)

**Table S4 Panel A (Friday minus Monday strategy returns of Amihud’s Illiquidity measure)**

|  | **Friday Long-Short** | **T statistics** | **Monday Long-Short** | **T statistics** | **Friday – Monday** | **T statistics** |
| --- | --- | --- | --- | --- | --- | --- |
| **CAPM** | .0116829 | (4.19) | -.0012540 | (-3.12) | .0129768 | (4.98) |
| **FF3** | .0101083 | (3.85) | -.0016920 | (-3.49) | .011801 | (4.39) |
| **Carhart4** | .0092849 | (3.66) | -.0018611 | (-3.61) | .011148 | (3.79) |
| **FF5** | .0088021 | (3.15) | -.0021998 | (-3.88) | .011002 | (4.62) |

**Table S4, Panel B (Friday minus Monday strategy returns of Bid-Ask Spread measure)**

|  | **Friday Long-Short** | **T statistics** | **Monday Long-Short** | **T statistics** | **Friday – Monday** | **T statistics** |
| --- | --- | --- | --- | --- | --- | --- |
| **CAPM** | .0063922 | (4.22) | .0017380 | (3.19) | .0046542 | (2.96) |
| **FF3** | .0071930 | (4.70) | .0016927 | (3.58) | .0055003 | (2..75) |
| **Carhart4** | .0075029 | (4.12) | .0013362 | (3.87) | .006167 | (3.28) |
| **FF5** | .0069203 | (4.09) | .0012947 | (4.02) | .0056256 | (3.15) |

Table S4 examines Friday minus Monday monthly portfolio returns based on Amihud’s illiquidity measure (Panel A) & Bid-Ask spread measure (Panel B) to invest in stocks with low institutional ownership on a particular day. The table reports the values of Alpha for CAPM, Fama& French 3 factor Model, Carhart 4 factor model, and Fama& French 5 factor model. Portfolios are equally weighted, and values of t-statistics are adjusted for autocorrelation & heteroscedasticity.

**
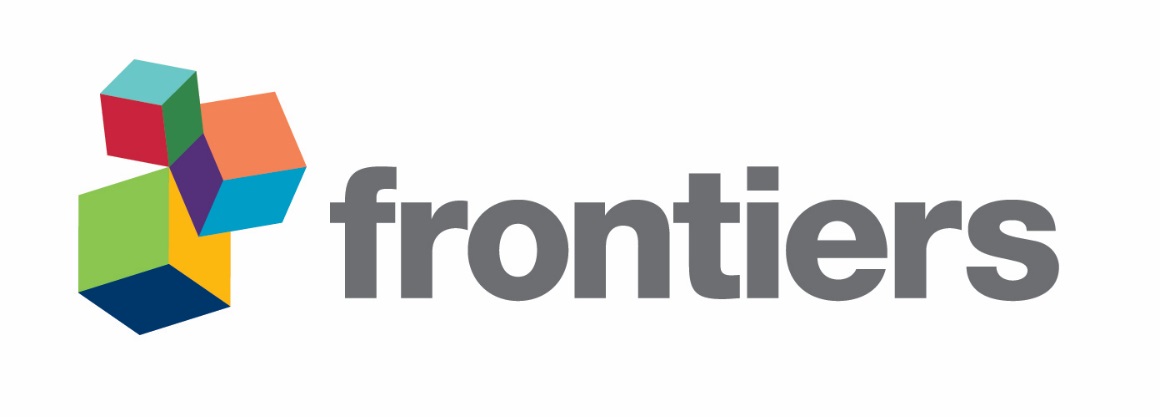
**
